# Environmental quality, functional control, and social design as determinants of perceived comfort and psychological well-being among older residents in Chinese continuing care retirement communities

**DOI:** 10.1186/s12877-026-07473-z

**Published:** 2026-04-21

**Authors:** Fengyu Xia, Tamilsalvi Mari, Sujatavani Gunasagaran

**Affiliations:** 1 The Design School, Taylor’s University, Selangor, Malaysia; 2 School of Architecture, Building and Design, Taylor’s University, Selangor, Malaysia

**Keywords:** Functional control, Perceived comfort, Psychological well-being, Older adults, CCRC, Environmental quality, Social design

## Abstract

This study investigates how the design of environmental quality, functional control, and social design affects the psychological well-being of older people living in Chinese Continuing Care Retirement Communities (CCRCs). The study uses concepts from environmental psychology and salutogenic design to suggest a mediation model in which perceived comfort is the link between spatial features and mental health outcomes. This study used structural equation modelling to investigate data from surveys of older people living in several CCRCs in eastern China. The results show that environmental quality greatly increases perceived comfort, and social design has a strong direct effect on both comfort and well-being. But perceived comfort didn't have a big effect on the link between design factors and psychological well-being. These results show how important social spatial design and environmental sensory quality are for the emotional lives of older residents. They also suggest that comfort alone may not be enough to improve mental health in institutional settings. The study has real-world effects on evidence-based design, policy, and future aging-in-place strategies in China.

**Supplementary Information:**

The online version contains supplementary material available at 10.1186/s12877-026-07473-z.

## Introduction

The global population is becoming older, and it is estimated that the percentage of the aged people over 60 years old is set to rise to 22 per cent in 2050 [36]. Social isolation, bereavement, decreasing functional capacity, and chronic illnesses are uniquely susceptible to older adults, which lead to increased vulnerability to depression and anxiety in old age [33]. These tendencies focus on the increasing significance of the environments that will not only meet the physical care needs but also provide active support to the psychological well-being and healthy ageing.

China experiences one of the fastest demographic changes in the world. The number of individuals above 60 years of age and the elderly will surpass 400 million by 2035, leading to a sudden increase in the demands of long-term and supportive housing [22]. As a reaction, CCRCs have grown at a very fast rate both publicly and privately. Although CCRCs are expected to facilitate the combination of housing services, healthcare, and daily support services, their potential to enhance the mental and emotional well-being of residents has not been researched, especially in the Chinese setting, where institutional care models and cultural considerations do not align with western countries [15].

Substantial studies on environmental psychology and evidence-based design indicate that the built environment can affect emotional states, stress levels, and psychological health. The building changes, including natural lighting, nature-related scenery, noise mitigation, and human-scale architectural planning, have been linked to the level of reduced stress and better mood of older adults [30, 40]. A smaller-scale, home-like setting and mutual social interaction with community facilities have been found to foster the independence of residents and their emotional well-being in long-term care facilities, with examples of such models being European dementia villages and the Green House Project in the United States [38]. The Chinese literature also indicates a positive relationship between access to green space and person-centered environments with life satisfaction and self-reported health, and a negative relationship with depressive symptoms in older adults [41, 42].

Despite these developments, several gaps in the literature still exist. Firstly, most investigations focus on environmental, functional, or social design aspects separately, without explaining how they vary conceptually or interrelate to influence the outcome of psychological processes. Second, there is a lack of empirical studies on CCRCs specifically in China, though the number of these types of communities is increasing at a high rate. Third, the psychological mechanisms by which spatial design affects mental health poorly are also unclear [21].

To fill these gaps, this paper conceptualises three categories of CCRC design factors, which are related to each other yet distinct. Environmental quality is the sensory and aesthetic attributes of the physical environment, such as lighting, colour, noise, exposure to nature, and artwork, which are the factor that influences the perceptual and emotional experience of residents. Functional control embodies the capacity of residents to mediate and subjectivise the immediate surroundings, i.e., to control light, temperature, furnishing, and spatial orientation, which are critical values of autonomy and competence based on the self-determination theory. Social design aims at spatial characteristics designed to facilitate social interaction, social support, and privacy, such as shared spaces, furnishings, and personal spaces, which are in line with social sustainability and psychosocial wellness theories.

The perceived comfort is central to this framework, which refers to the subjective experience of residents of physical comfort, emotional safety, and the psychosocial support of them in the place where they live. According to previous literature, perceived comfort is a combination of sensory satisfaction, sense of safety, autonomy, and social connectedness, and thus, it may be a viable route through which spatial design can affect psychological well-being [32, 39]. Nevertheless, perceived comfort has not been empirically studied as an intermediate process in research on institutional living conditions, especially in Chinese CCRCs.

This study proposes and tests a mediation model, whereby psychological well-being is directly and indirectly affected by the environmental quality, functional control, and social design via perceived comfort, through referencing environmental psychology, salutogenic design, and self-determination theory. The study improves the current literature in the field in three ways, first, it clarifies the unique and combined role of environmental, functional and social design factors in influencing the mental well-being of older adults and second, it enhances current theoretical knowledge by empirically testing the perceived comfort as a psychological process that connects spatial design with the well-being of older adults and finally, the research informs the design, planning and policy formulation processes of CCRCs in China and thus promotes more holistic and psychologically responsive treatment of older adult environments.

## Literature review

### Theoretical support

**Fig. 1:**
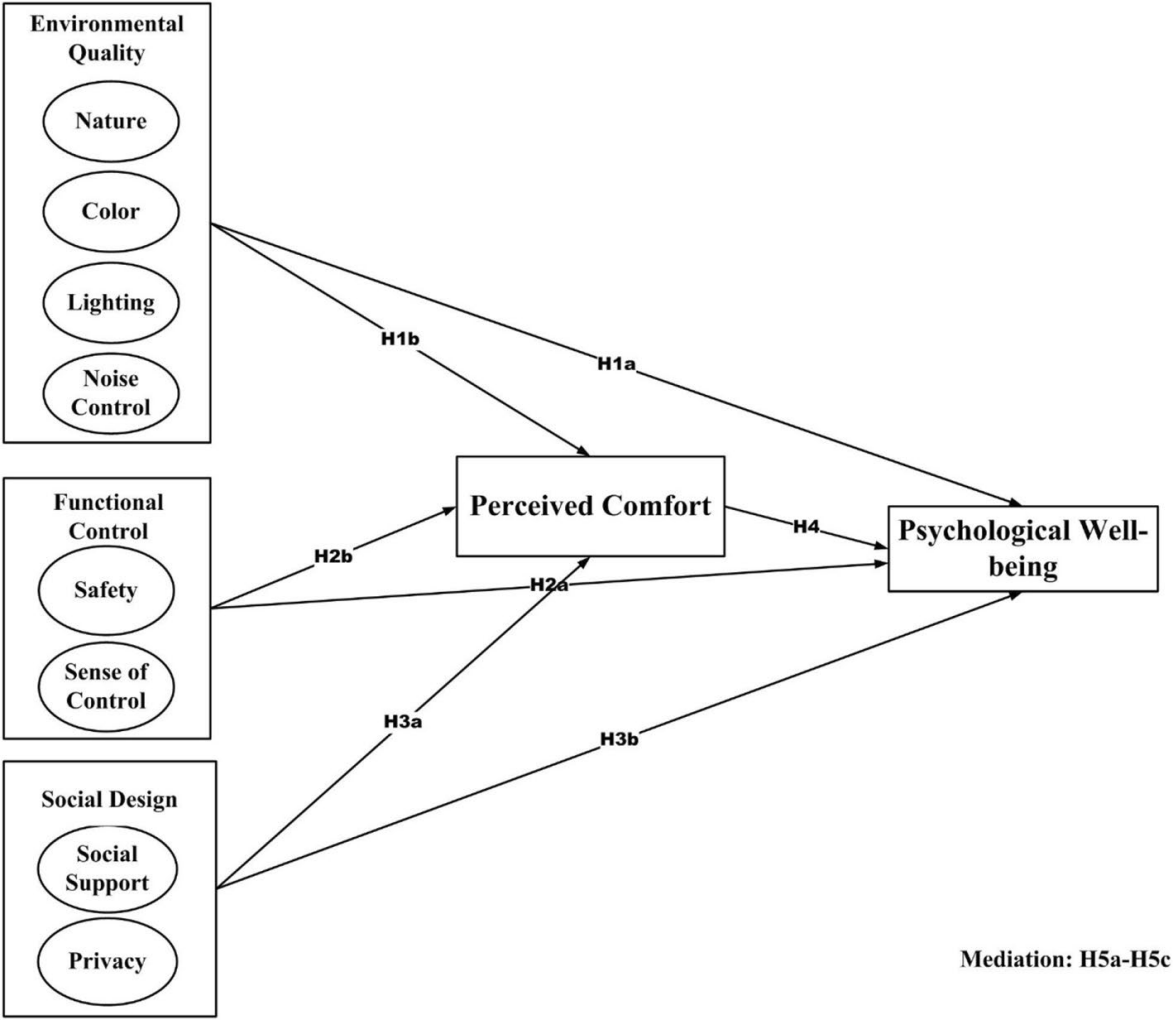
Conceptual framework

Three distinct theoretical perspectives support the study's conceptual foundation. First, Environmental Psychology asserts that the physical environment has a big effect on how people act, think, and feel [11]. Natural elements, clear spaces, and sensory regulation are known to lower stress and improve the perceived quality of the environment, especially for older adults living in their own homes [30]. Second, Self-Determination Theory (SDT) contends that autonomy, competence, and relatedness are important psychological needs for health [7]. In this case, design elements that improve functional control, like personal adjustability, safety, and spatial predictability, help residents feel more independent and in charge of their lives, which is especially important for older people. Third, Salutogenic Design Theory, which is based on Antonovsky's idea of the sense of coherence, explains that environments that make things easier to understand, manageable, and meaningful are good for mental health [2]. The idea of "perceived comfort" in this study combines these theoretical ideas by capturing the resident's personal experience of environmental harmony, control, and social support. These frameworks work well together to explain how design-driven interventions can improve the mental health of people living in CCRCs. Figure 1 provides a conceptual framework.

### Psychological well-being in aging populations

As the world's population gets older, psychological well-being has become an important part of healthy ageing. For older adults, psychological well-being includes emotional stability, a sense of purpose, independence, and social connectedness. All these factors are linked to better health outcomes and quality of life [27]. Older people who live in places like CCRCs are especially at risk for loneliness, depression, and anxiety because of changes in their social roles, health, and independence [29]. So, making places that are good for mental health is not only a clinical priority, but also a challenge for architects and policymakers.

### Environmental design and mental health

Environmental psychology is a way of thinking about how physical spaces affect mental states. Ulrich [30] and [18] did classic studies that showed that natural elements, daylight, and low-stress environments lower both physical and mental stress a lot. Built environments that are easy to get to, look good, and are easy to understand have been shown to help older people control their emotions, calm down, and feel better[4]. Recent studies in care settings show that design changes like more natural light, better sound control, and access to plants can improve residents' mental health and even lower their need for medication [38].

### Perceived comfort as a mediating construct

Perceived comfort is a person's own opinion of how easy, safe, and peaceful a physical space is. It is a response to things in the environment, like temperature, light, noise, and how things are arranged in space. It is both a physical and mental response. It is becoming more and more clear that it is a key factor in older people's health [1]. According to environmental psychology, perceived comfort includes not only physical ease but also emotional and social factors like safety, privacy, and familiarity [25]. Researchers have found that environments that people think are more comfortable can lower stress, lift mood, and make it easier to interact with others, especially in residential care settings [24].

### Environmental quality

Environmental quality is the sensory and aesthetic state of a space. It includes events like natural light, air quality, thermal comfort, sound quality, and how easy it is to see nature. These features are essential for making places that help people heal, and they are linked to less stress, better sleep, and better control of emotions [19]. These factors are even more important for older adults, who often spend a lot of time inside. Research in care facilities has shown that being outside and getting natural light can help with depression and cognitive decline [34].

### Functional control

Functional control focusses on how well residents can move around, customise, and take care of their immediate surroundings. For example, they can change the lighting, open and close windows, or choose where to sit. Functional control is based on Self-Determination Theory and helps people feel like they have control over their lives, which is a well-known part of mental health [28]. Older people who can control their own space say they are happier with their lives and less anxious [16]. In CCRC settings, ergonomic furniture, adjustable lighting, and room layouts that are easy to get to are all features that help with functional control.

### Social design

Social design includes both physical and programmatic elements that make it easier for people to interact with each other, feel safe, and have privacy. These are communal lounges, semi-private outdoor spaces, and areas that can be used for both group and individual activities. Research in residential care shows that well-designed social spaces help people connect, feel less lonely, and feel like they are part of a community, all of which are important for mental health as people get older [20]. In collectivist cultures like China, where social harmony and modesty affect how people see space, the right amount of public and private space is very important [35].

### Hypotheses development

#### Environmental quality and psychological well-being

Research has shown that environmental quality, such as natural light, plants, colour, sound comfort, and air quality, can improve mental health, especially in older people who spend more time indoors. Daylight and views of nature are two things that help lower stress and keep your emotions in check. Sensory-rich and beautiful environments in residential care settings have been linked to less depression and better mood [3, 10].

H1a: *Environmental quality is positively associated with psychological well-being among older residents in CCRCs.*

CCRCs provide enough daylight, use sympathetic color combinations, provide views of natural scenery, and incorporate effective acoustic reduction initiatives that will lead to physical comfort and emotional relaxation as important attributes of perceived comfort [40]. Salutogenic design theory also gives greater emphasis to the importance of the environments that support coherence and sensory equilibrium in enhancing the ability of individuals to handle stress and maintain well-being [12]. It is, therefore, expected that as the quality of the environment in CCRCs improves the higher the perceptions of comfort among the elderly residents.

H1b: *Environmental quality is positively associated with perceived comfort among older residents in CCRCs.*

#### Functional control and psychological well-being

Functional control is how well residents can safely and independently interact with their living space. Self-Determination Theory says that features that give people more freedom, like adjustable lighting, clear layouts, and safe navigation, help people feel like they have control over their lives and can do things. These are important for well-being [19]. People who feel like they have control over their lives in long-term care settings have been shown to have less anxiety and more satisfaction with their lives [23].

H2a: *Functional control is positively associated with psychological well-being among older residents in CCRCs.*

In older adults, because the CCRCs are occupied with older adults, ease of living is provided by the fact that the elderly can customize environmental parameters to suit their physical abilities, their preferences, and their daily activities [8]. Adaptive lighting and easily accessible controls, clear wayfinding signs, and room customization opportunities alleviate helplessness and institutionalization. In the person-centered care approach, the outlook on an environment where functional autonomy is supported will be more prone to be interpreted as accommodating and supportive, as opposed to restrictive [19]. In line with this, increased functional control in CCRCs will likely result in improved perceived comfort among the residents.

H2b: *Functional control is positively associated with perceived comfort among older residents in CCRCs.*

#### Social design and psychological well-being

Social design focuses on spatial arrangements that encourage interaction, companionship, and privacy, which are important social needs for older adults. Well-planned common areas can help people feel less alone and more emotionally strong. On the other hand, places that don't offer chances for social integration are linked to worse mental health outcomes [4].

H3a: *Social design is positively associated with psychological well-being among older residents in CCRCs.*

Social elements of social design, such as accessible communal lounges, flexible furniture, and personal areas of meaningful interaction in CCRCs, help residents to sustain social identities and supportive relationships. The existing body of research suggests that social interaction enhances a less lonely environment as well as leads to the creation of a more comfortable and home-like experience [31]. Notably, the availability of social and personal spaces allows residents to control the social exposure according to their own needs, which promotes the perceived comfort even more. Therefore, social design is likely to be positively associated with older residents' perceived comfort.

H3b: *Social design is positively associated with perceived comfort among older residents in CCRCs.*

#### Perceived comfort and psychological well-being

Perceived comfort refers to the subjective assessment of physical, emotional, and psychological comfort by an individual in their place of residence. Environmental psychology and salutogenic theory-based theoretical frameworks assume that comfortable environments mitigate the effects of chronic stress and lead to emotional control, which benefits psychological well-being [31]. Relational comfort, such as a sense of safety, relaxation, and comfort, is especially sensitive to older adults, and they may be more susceptible to anxiety, depression, and stress-related conditions [33]. The Ryff model of psychological well-being has focused on environmental mastery, autonomy, and positive relations, which can be enhanced by comfortable living conditions [27]. Perceived comfort can be a proximal state of psychology in CCRCs where the environment can induce greater results in well-being. When the residents report physical relaxation, emotional security, and satisfaction with the environment, chances are high that they will report positive affect, life satisfaction, and psychological stability. As a result, psychological well-being among older residents is likely to be positively linked to perceived comfort.

H4: *Perceived comfort is positively associated with psychological well-being among older residents in CCRCs.*

#### Perceived comfort as a mediator

Perceived comfort refers to the level of comfort, safety, and emotional satisfaction a resident experience in their home. It combines both physical and psychological factors, and it may be the way that design factors affect psychological outcomes [37]. Past studies have shown that comfort affects how environmental factors affect satisfaction, emotional security, and stress management [17].

H5a: *Perceived comfort mediates the relationship between environmental quality and psychological well-being.*

H5b: *Perceived comfort mediates the relationship between functional control and psychological well-being.*

H5c: *Perceived comfort mediates the relationship between social design and psychological well-being.*

### Conceptual framework

## Methods

### Questionnaire development

The questionnaire was designed to examine the association between design factors in Chinese CCRCs and elderly residents’ psychological well-being. It was divided into four sections and employed a standardized Likert-type response format throughout.

Section A collected demographic information, including age, gender, marital status, education level, health status, length of stay, and living arrangement. Section B utilized the CCRC design factors (Environmental Quality, Functional Control, and Social Design) to evaluate residents’ experiences of healing spaces within the CCRC environment [5, 31]. Section C employed the Perceived Comfort Scale tailored for the context of mental health among older residents in Chinese CCRCs, drawing from established healthcare environment research such as the Perceived Hospital Environment Quality Indicators (PHEQIs) (e.g., “spatial-physical comfort”[31] subscale) [5]. Section D includes 18 items of the Ryff Short Form for the Six Dimensions (autonomy, environmental mastery, personal growth, positive relations with others, purpose of life) of Psychological Well-Being [26].

Next, the scale was translated using the back-translation method. A professional translator translated the draft into Chinese. Subsequently, a different professional translator back-translated it from Chinese to English. The researchers then compared the original English items with the back-translated items, resulting in the development of the first draft of the questionnaire.

### Research sites, sampling, and participants

**Fig. 2:**
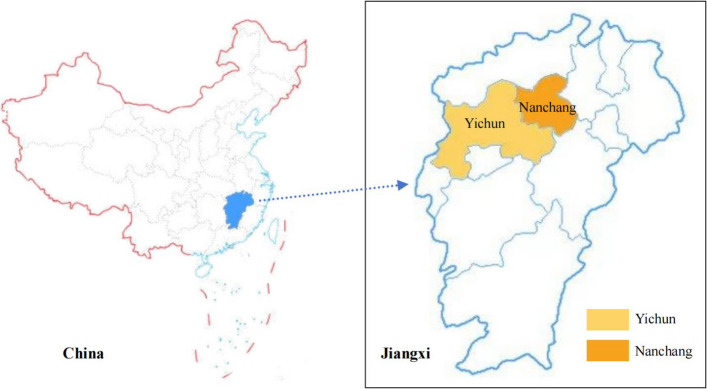
The geographical location of the research sites

**Fig. 3:**
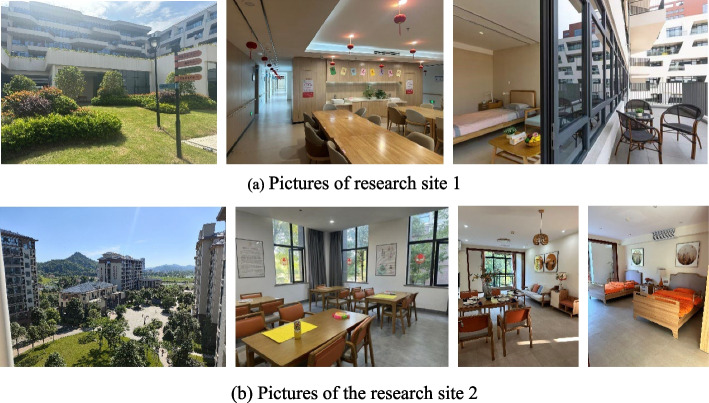
Pictures of research sites

This study was conducted at two CCRCs in Jiangxi Province: Research Site 1 in Nanchang City and Research Site 2 in Yichun City (as shown in Fig. 2). Jiangxi Province was selected due to its rapid aging population and significant government efforts to support elderly care services, making it an ideal context for examining healing spaces in CCRCs. The selected CCRCs are recently established, high-quality facilities with similar environmental characteristics, including comprehensive healing space design factors (Fig. 3). Their comparable quality ensures consistency in data collection and allows for reliable analysis of the association between healing spaces and the mental health of elderly residents. The site selection process involved an extensive review of CCRCs across Jiangxi Province, followed by direct communication with facility management to confirm their operational status. On-site observations were then conducted using a developed checklist to assess the presence of key design factors. Despite initial challenges in gaining access to one shortlisted facility, the two selected CCRCs fully met the research criteria, ensuring the feasibility and robustness of the study.

Purposive sampling was used to select respondents who fulfilled the following inclusion criteria:

- (1) aged 60 years or older.
- (2) a one-month stay at the community-based retirement center (CCRC).
- (3) sufficient cognitive and physical ability to understand and answer the questionnaire, as confirmed by CCRC employees and observations made by the researcher.

Even though purposive sampling limits statistical generalizability, it can be explained by the studies that are aimed at the evaluation of the experiential components of specialised gerontological environments like CCRCs. A variety of age groups, genders, and living accommodations were used to attract participants to make the sample more representative. The demographic structure of the sample was consistent with the information of local census on institutionalized older adults in Jiangxi Province, especially the age and sex distribution.

A total of 205 elderly residents from the two CCRCs participated in this study. At research site 1, 97 respondents who agreed to participate in the survey were recruited. Similarly, at research site 2, 108 respondents who agreed to participate in the survey were recruited. Table 1 provides the demographic characteristics of the participants. The mean age of the participants in this study was 80.3 years. Table 1 shows the descriptive characteristics of participants.

**Table 1:** Descriptive characteristics of participants

Characteristics	Frequency	Percentage (%)
Age (years)		
Below 60	0	0
60–69	12	5.85
70–79	76	37.07
80–89	104	50.73
90 and above	13	6.34
Gender		
Male	93	45.37
Female	112	54.63
Marital status		
Single	0	0
Married	43	20.98
Divorce	0	0
Widowed	162	79.02
Level of education		
No education	4	1.95
Elementary school	13	6.34
Middle school	56	27.32
High school	80	39.02
University	52	25.37
Master's degree or higher	0	0
Health status		
Excellent	9	4.39
Good	110	53.66
Fair	78	38.05
Poor	8	3.90
Length of residence		
Less than half a year	50	24.39
More than half a year	155	75.61
Living arrangement		
Alone	145	70.73
With spouse/partner	31	15.12
With other residents	29	14.15

Table 2 shows descriptive data for the primary study constructs, which are Environmental Quality, Functional Control, Social Design, Perceived Comfort, and Psychological Well-Being. Five-point Likert scales were used to measure all the variables. The means and standard deviations were good, which suggests there was enough variation and the data were good for structural equation modeling.

**Table 2:** Descriptive statistics of study variables

Construct	Scale Range	Mean	SD
Environmental Quality	1–5	3.87	0.62
Functional Control	1–5	3.65	0.68
Social Design	1–5	3.92	0.59
Perceived Comfort	1–5	3.88	0.61
Psychological Well-Being	1–5	3.74	0.57

### Data collection procedure

Paper survey questionnaires were used to collect data from elderly residents in the two selected Chinese CCRCs. A representative group of these residents was invited to complete the questionnaires through face-to-face interviews conducted in December 2024. The first author, accompanied by the research assistant and previously engaged staff who assisted in pilot testing, distributed paper questionnaires to representative respondents at each research site. During this process, they explained the purpose of the questionnaire and the study to the potential participants, providing them with an information sheet and a consent form for review.

### Data analysis

PLS-SEM was performed to assess the measurement model and structural model, including the testing of hypotheses. In a complex research model, SEM can be used to calculate multiple paths [13].

#### Measurement model

Table 1 shows that the measurement model assessment is very reliable and valid for all constructs in both China and Malaysia. The values for Cronbach's Alpha range from 0.85 to 0.91, which means that each construct is very consistent with itself. The Composite Reliability (CR) values are higher than the suggested level of 0.70, which further supports the reliability of the construct. The Average Variance Extracted (AVE) values, which range from 0.63 to 0.72, are higher than the 0.50 benchmark. This supports convergent validity, which means that the items accurately measure their respective constructs. The Heterotrait-Monotrait (HTMT) ratio, which shows that all values are below the 0.85 threshold, also confirms discriminant validity, which means that the constructs are different from each other in a real way. Table 3 explains the discriminant validity, while Table 4 provides the descriptive analysis results.

**Table 3:** Discriminant validity

Constructs	ENV-Q	FC	PC	SC	PWB
Environmental Quality (ENV-Q)					
Functional Control (FC)	0.036				
Perceived Comfort (PC)	0.128	0.056			
Social Design (SC)	0.031	0.080	0.121		
Psychological Well-Being (PWB)	0.040	0.031	0.026	0.120	

Environmental Quality (ENV-Q), Functional Control (FC), Social Control (SC), Perceived Comfort (PC), Psychological Wellbeing (Psy-wellbeing)

**Table 4:** Descriptive analysis results

Construct/Associated Items	Factor loadings	CR	AVE
Functional Control		0.96	0.877
Sense of control (SC)			
1. Able to open and close doors	0.892		
2. Able to control the position and orientation of the bed	0.875		
3. Able to adjust the lighting intensity	0.860		
4. Able to control the volume of the TV and music	0.894		
5. Able to control the room temperature and humidity	0.854		
6. Able to open or close windows	0.874		
7. Able to control the call button	0.871		
8. Able to easily find your way or destination	0.879		
9. Able to personalize your room (e.g. placing family photos)	0.834		
Safety (SA)			
1. Having handrails (e.g. having bars in the bathroom/toilet of your room)	0.853		
2. Having proper non-slip flooring	0.856		
3. Having a safe storage area to stow personal belongings	0.851		
4. Having proper arrangement and location of furniture, clear and uncluttered pathways	0.862		
5. Having visual connection between rooms and nursing stations	0.851		
Social Design		0.97	0.89
Social support (SS)			
1. Having areas to facilitate communication and interaction (e.g. social activities and entertainment areas)	0.785		
2. Having comfortable and movable furniture (e.g. comfortable and movable chairs)	0.786		
3. Having areas for families and friends to stay overnight	0.794		
Privacy (PR)			
1. Having a single bedroom	0.827		
2. Having areas for private conversation	0.820		
3. Having visual privacy (e.g., having curtains and partitions)	0.851		
Environmental Quality		0.96	0.88
Colour (CO)			
1. Bright colours (e.g. light green, light yellow, white)	0.885		
2. Natural colours (e.g. colour of plants, sky, earth and sea)	0.911		
3. Warm colours (e.g. red, orange, yellow)	0.869		
4. Cool colours (e.g. green, blue, purple)	0.909		
5. Vibrant colours (e.g. vivid blue, purple)	0.866		
6. Harmonious colours (e.g. monochromatic, complementary, analogous, and split complementary colors)	0.869		
7. Chinese traditional colours (e.g. the following 12 Chinese traditional colours)	0.902		
Nature (NA)			
1. Having natural materials (e.g. wooden flooring and furniture)	0.749		
2. Having natural sounds (e.g. sound of birds, water, breeze)	0.819		
3. Having water features (e.g., fountains, ponds, waterfalls)	0.856		
4. Having fresh air	0.778		
5. Having access to nature like flowers, plants, and trees	0.783		
6. Having window views of nature such as flowers, plants, trees, and the sky	0.734		
7. Having a view of nature	0.743		
Art (AR)			
1. Having nature-based artwork (e.g. paintings or photographs of natural scenery)	0.839		
2. Having areas for art viewing (e.g. areas for painting, calligraphy exhibitions)	0.847		
3. Having areas for artistic activities (e.g. areas for dancing, singing, painting)	0.814		
4. Having Chinese culture-relevant artworks (e.g. Chinese traditional painting, traditional patterned porcelain, and paper cutting)	0.816		
Lighting (LI)			
1. Having adequate artificial light	0.744		
2. Having plenty of daylight	0.782		
3. Having low-positioned sensor night lights along the route used for getting up at night	0.783		
Noise control (NC)			
1. Your room is removed from noise-producing areas	0.794		
2. Using silent machines in your room	0.781		
3. Your room is soundproofed	0.838		
Perceived Comfort		0.98	0.89
1. My living space feels calm and peaceful	0.853		
2. I can move around my private and communal spaces without difficulty	0.856		
3. The temperature in my room is comfortable throughout the day	0.851		
4. I am satisfied with the amount of natural light entering my room	0.862		
5. The furniture provided is comfortable and meets my physical needs	0.851		
6. The overall noise level allows me to rest and feel relaxed	0.881		
7. I feel physically safe in both my room and shared areas	0.785		
8. The colors and interior design help me feel emotionally at ease	0.786		
9. I have adequate personal space to maintain privacy and comfort	0.794		
10. The environment helps me feel emotionally stable and less anxious	0.853		
11. Bathroom and hygiene facilities are clean and refreshing	0.862		
12. I can control aspects of my room environment (e.g., lighting, ventilation)	0.851		
13. Communal areas (e.g., lounges, gardens) feel inviting and homely	0.881		
14. I enjoy spending time in the physical surroundings of this community	0.785		
15. The environment helps me feel connected to nature or the outdoors	0.786		
16. Overall, this place feels like a comfortable home to me	0.889		
Psychological Well-Being Scale		0.97	0.90
1. I am not afraid to voice my opinions, even when they are in opposition to the opinions of most people	0.862		
2. My decisions are not usually influenced by what everyone else is doing	0.851		
3. I tend to worry about what other people think of me. *(reverse)*	0.881		
4. In general, I feel I am in charge of the situation in which I live	0.785		
5. The demands of everyday life often get me down. *(reverse)*	0.786		
6. I am quite good at managing the many responsibilities of my daily life	0.862		
7. I think it is important to have new experiences that challenge how you think about yourself and the world	0.851		
8. I have a sense that I have developed a lot as a person over time	0.881		
9. I do not enjoy being in new situations that require me to change my old familiar ways of doing things. *(reverse)*	0.785		
10. People would describe me as a giving person, willing to share my time with others	0.786		
11. I have not experienced many warm and trusting relationships with others. *(reverse)*	0.862		
12. Maintaining close relationships has been difficult and frustrating for me. *(reverse)*	0.851		
13. Some people wander aimlessly through life, but I am not one of them	0.862		
14. I have a sense of direction and purpose in life. *(if original short form)*	0.851		
15. I sometimes feel as if I’ve done all there is to do in life. *(reverse)*	0.881		
16. I like most parts of my personality	0.785		
17. When I look at the story of my life, I am pleased with how things have turned out so far	0.851		
18. In many ways I feel disappointed about my achievements in life. *(reverse)*	0.881		

#### Structural model

After determining that the measurement model was reliable and valid, the overall model fit and explanatory power of the PLS-SEM were evaluated. The Standardized Root Mean Square Residual (SRMR) was employed as the main universal goodness-of-fit metric, while coefficients of determination (R^2^ and modified R^2^) were given to evaluate the model's explanatory capability for endogenous constructs. Table 5 presents the model fit and explanatory power indices.

**Table 5:** Model fit indices of the measurement model

Fit Index/Construct	Value	Recommended Threshold	Decision	References
SRMR	0.062	< 0.08	Acceptable	Hu and Bentler [14]
R^2^ (Perceived Comfort)	0.036	≥ 0.02 (small effect)	Acceptable	Chin [6]
Adjusted R^2^ (Perceived Comfort)	0.025	–	–	
R^2^ (Psychological Well-Being)	0.016	≥ 0.02 (exploratory)	Acceptable	Falk and Miller [9]
Adjusted R^2^ (Psychological Well-Being)	0.000	–	–	

The assessment of the structural model (Fig. 3) in this study involved generating related values of the path coefficient (*β*) and the corresponding t-values. This study performed 5,000 bootstraps random resamplings to calculate the t-values. The results of the structural model show that Environmental Quality has a statistically significant positive effect on perceived comfort (β = 0.125, *p* = 0.02). This shows how important design features like natural light, sound control, and visual appeal are in making residents feel comfortable in CCRCs. However, neither environmental quality (*p* = 0.71) nor functional control (*p* = 0.56) has a direct significant effect on psychological well-being. Also, Perceived Comfort does not significantly predict well-being (*p* = 0.96). Interestingly, social design is a strong positive predictor of both perceived comfort (β = 0.126, *p* = 0.05) and psychological well-being (β = 0.119, *p* = 0.05). This suggests that residents see communal spaces and chances to interact with others as helpful and emotionally supportive. These results show how important social spatial design is for improving mental health in elder care settings, even when comfort doesn't seem to play a role in this relationship. The results suggest that social design may have more than just comfort benefits; it may also have direct functional and psychosocial benefits. Tables 6 & 7 explain the structural model and R-square results.

**Table 6:** Structural model

Hypotheses		Original sample (O)	Sample mean (M)	Standard deviation (STDEV)	T statistics (|O/STDEV|)	P values
H1a	ENV-Q—> Psy-wellbeing	0.028	0.028	0.075	0.368	0.71
H1b	ENV-Q—> PC	0.125	0.127	0.054	2.317	0.02
H2a	FC—> Psy-wellbeing	0.043	0.041	0.075	0.574	0.56
H2b	FC—> PC	0.081	0.070	0.078	1.040	0.29
H3a	SC—> Psy-wellbeing	0.119	0.123	0.064	1.847	0.05
H3b	SC—> PC	0.126	0.125	0.070	1.816	0.05
H4	PC—> Psy-wellbeing	0.003	−0.001	0.063	0.043	0.96

Environmental Quality (ENV-Q), Functional Control (FC), Social Control (SC), Perceived Comfort (PC), Psychological Wellbeing (Psy-wellbeing)

**Table 7:** Coefficient of determination (R^2^)

Endogenous Construct	R^2^	Adjusted R^2^
Perceived Comfort (PC)	0.036	0.025
Psychological Well-Being	0.016	0.000

Table 6 shows the coefficient of determination (R^2^) for the constructions that are endogenous. The model accounts for 3.6% of the variance in Perceived Comfort and 1.6% of the variance in Psychological Well-Being, demonstrating limited yet adequate explanatory power for exploratory research investigating intricate psychosocial and environmental interrelations.

#### Mediation results

The mediation analysis shows that Perceived Comfort does not significantly mediate the relationship between any of the three design factors and Psychological Well-being because it does not have a significant direct effect on well-being (β = 0.003, *p* = 0.96). Environmental Quality does make people feel more comfortable (β = 0.12, *p* = 0.02), but it doesn't have a direct effect on well-being through comfort because there isn't a strong link between comfort and well-being. In the same way, neither Functional Control nor Social Design has mediated effects through comfort. However, Social Design does have a direct significant effect on both Perceived Comfort and Psychological Well-being (*p* = 0.05). These results suggest that Perceived Comfort does not work as a mediator in the current model. Instead, the psychological benefits of Social Design may come from other things, like better social interaction, emotional support, or a sense of belonging, rather than just comfort. Table 8 explains the mediation results.

**Table 8:** Mediation results

		Direct Effect β (p)	Indirect Effect via PC β (p)	Total Effect β
H5a	ENV—> PC—> wellbeing	0.028 (0.713)	0.000 (0.968)	0.028
H5b	FC—> PC—> wellbeing	0.043 (0.566)	0.000	0.043
H5c	SC—> PC—> wellbeing	0.119 (0.050) *	0.000 (0.970)	0.119

Environmental Quality (ENV-Q), Functional Control (FD), Social Control (SC), Perceived Comfort (PC), Psychological Wellbeing (Psy-wellbeing)

## Discussion and theoretical contributions

### Discussion

This research study explored how environmental quality, functional control, and social design affect psychological well-being in older residents in CCRCs, and perceived comfort is one of the purported mediating variables. The findings offer subtle information that goes beyond current knowledge in environmental psychology and gerontology by describing the mechanisms of space that have the greatest impact on the mental health of people in institutional ageing contexts and by explaining why certain put-forward hypotheses do not come about as hypothesised.

#### Environmental quality, comfort, and the limits of sensory-based explanations

In line with previous literature in environmental psychology and other studies involving healing as well as environmental studies, the environmental quality showed statistically significant positive correlation with perceived comfort [41]. Design characteristics like natural lighting, acoustic control, visual harmony and access to nature seem to be useful in reducing the stress on the senses and contributing to the overall feeling of subjective comfort, which supports the classical theories of restorative-environment [18]. However, the influence of environmental quality was not a direct one on the psychological well-being, neither was it mediating this relationship through perceived comfort. This result suggests a significant hypothetical correction: sensory and aesthetic comfort can be required, but not enough to provide more psychological well-being in long-term institutional contexts. In contrast to acute clinical settings, CCRCs represent long-term residential settings where material conditions are not the only determinants of well-being, but long-term psychosocial processes, the pursuit of meaning, a feeling of belonging, and social role continuity are also facilitated [40]. Therefore, though environmental quality will increase temporary comfort, its psychological gains could decrease as time progresses unless this comfort is supplemented by the possibilities of socialization and meaningful activity.

#### Functional control, autonomy, and institutional contexts

Contrary to the expectations based on self-determination theory, functional control did not have a significant influence on perceived comfort and psychological well-being. This conclusion does not deny the core significance of autonomy per se, but rather it pre-empts the situational constraints of environmental regulation in institutional care conditions (Li-Hsing and Chia-Chan 2020). Contrasts in CCRCs' control avenues can be operationalized or limited by operational procedures, safety regulations, and care procedures, and this may dilute the perception of control as a salient source of comfort or psychological good. Theoretically, the findings indicate that autonomy in institutional settings later-in-life can be relatively or interpersonally experienced more than instrumentally [41]. Instead of suggesting exclusive dominance of physical attributes, the elderly might gain some feeling of autonomy out of social activity, decision-making in communal life, and relational respect, aspects that are poorly measured in traditional functional-control scales. In line with this, the results would narrow the application of self-determination theory in eldercare settings by highlighting the importance of context-specific manifestations of autonomy.

#### Social design as a primary pathway to psychological well-being

The most prominent observation of the study is that the direct influence of social design on perceived comfort and psychological well-being is significant. Aspects of space that support social engagement, create the possibility of companionship, and provide a balance between privacy and accessibility are quite apparent to the mental health of inhabitants [28]. Notably, this effect was significant upon adjusting the perceived comfort, which suggests that social design is a well-being contributor based on mechanisms other than those grounded in physical or sensory comfort. This outcome resonates well with the relatedness dimension of self-determination theory and the socio-emotional selectivity theory, which focuses on the increasing salience of emotionally significant relationships in adulthood [20]. Social design seems to be a psychosocial asset, which promotes a sense of belonging, emotional stability, and identity persistence- the primary predictors of psychological health during later adulthood.

#### Why perceived comfort did not mediate design–well-being relationships

One of the primary contributions of this research is the explanation of the lack of mediator action of perceived comfort among design factors and psychological well-being. Even though comfort has been traditionally viewed as a proximal result of environmental quality, the current results indicate that comfort per se does not result in improved psychological well-being in CCRCs. A probable cause is that psychological well-being is conditional on higher-order assessments of meaning, social integration, and emotional fulfillment, none of which have a direct relationship with physical ease [16, 25]. Perceived comfort, in this place, therefore, may be a baseline environmental condition, a necessary but passive condition, which is a necessary but passive basis of mental-health outcomes and not an active mechanism. As soon as the threshold of comfort has been reached, additional improvement of well-being can be more sociologically and emotionally oriented, meaning active and relational. This interpretation makes the low variance explained in psychological well-being clear and explains why mediation is not shown, thus filling a timely gap in previous studies, which frequently assume linear comfort-well-being relationships.

#### Cross-cultural interpretation: collectivism and social meaning in Chinese CCRCs

The results are also interpreted in terms of a cross-cultural perspective. Well-being later in life is interconnected with social harmony, interdependence, and relational belonging, which in the Chinese collectivist context is highly associated with well-being. Older adults might place more importance on community living, community spaces, and social exposure than on individual comfort and privacy as it is understood in Western CCRC models. In turn, the social design elements will be more likely to appeal to culturally ingrained ageing, dignity, and emotional safety expectations. This cultural orientation, in part, could be the reason why the effects of social design on psychological well-being were greater than the effects of environmental quality or functional control. It further implies that models of healing environment developed in the West must be adjusted in non-Western institutional settings. Through an empirical illustration of this trend, the paper provides new cross-cultural information to the field of environmental gerontology and ageing-in-place studies.

### Theoretical contributions

This work contributes to the body of literature on environmental psychology, environmental gerontology, and age-friendly design with some salient theoretical contributions by refining the processes by which the built environment affects the psychological wellness in long-term residential care facilities, especially in a non-Western context.

#### Reframing the role of perceived comfort in environmental psychology

*First*, the current study contributes to the environmental psychology theory by critically revisiting the concept of perceived comfort as a psychological mediator between spatial design and well-being. Although earlier research has generally assumed the conceptualisation of comfort as an intermediate variable where the environmental factors influence mental health, the current evidence shows that the perceived comfort does not always result in increased psychological well-being among the elderly in community-based senior CCRCs. This is problematic to the implicit assumption of a linear comfort well-being path and implies that comfort can be an encoding of a baseline environmental condition as opposed to a dynamic psychological agent in long term institutional life. The study demonstrates the non-significant mediating effect of perceived comfort by means of empirical evidence, thus shedding a little more light on the internalisation of environmental experiences across time. It also differentiates between experiential comfort (comfort) and eudaimonic well-being, including purpose, control, and significant social relationships, which refines the existing models of environmental psychology by highlighting the possibility of purpose, control, and meaningful social relationships being insufficient to support deeper psychological well-being in residential ageing scenarios.

#### Extending self-determination theory to institutional ageing environments

*Second*, the study advances self-determination theory by contextualising its two key psychological needs, autonomy and relatedness, in institutional care settings. Although SDT historically considers autonomy because of control and choice, the non-significant impact of functional control allows one to propose that instrumental autonomy is limited or repackaged in CCRCs. Aging residents might also enjoy a condition of autonomy more in relational and social aspects, as well as in the participatory aspects of daily life, rather than direct control over the physical environment. On the contrary, the direct impact of social design on psychological well-being is strong, which supports the role of relatedness in adulthood. Spatial arrangements that accommodate social interaction, shared experiences, and emotional attachment seem to fulfill basic psychological requirements more than purely functional or sensory aspects of design. The result is an extension of SDT since it reveals that the salience of psychological needs can change during later adulthood and institutional contexts, and relatedness turns out to be a factor contributing to well-being as well as environmental control.

#### Advancing environmental gerontology through a multidimensional design framework

*Third*, the current study is relevant to environmental gerontology because it empirically separates the varying impacts of environmental quality, functional control, and social design into the same integrative paradigm. Most of the past studies tended to view these dimensions separately or as interchangeable dimensions of healing environments. By conducting tests on their unique pathways, the research demonstrates that social design is one of the major psychosocial pathways towards psychological well-being, and environmental quality is the main factor in increasing perceived comfort, not psychological well-being. Such a difference takes theory to a higher level of specificity, beyond general statements suggesting that the better the environment, the more mental health, and into a more detailed explanation of which specific dimension of the environment would be most relevant and why. It also answers the calls of more theory-based, mechanism-based research in ageing-in-place and institutional care research studies.

#### Contributing a cross-cultural perspective to CCRC research

Lastly, the paper has provided a valuable cross-cultural lesson by placing the CCRC design in the Chinese context of collectivism. The majority of the existing CCRC and healing environment theories have Western individualistic assumptions with a focus on privacy, independence, and personal control. The evidence of this study indicates that in the case of the Chinese elderly, social connectedness, communal life, and relational harmony are stronger determinants of psychological well-being than either personal comfort or control of the environment. This paper offers culturally sensitive information that questions the applicability of Western-based environmental design models through presenting the evidence of the preeminence of social design in a Chinese CCRC setting. It highlights the importance of culturally sensitive theories of age-friendly environments that consider social values, family norms, and collective identities in the construction of environmental experiences and mental health outcomes.

### Policy recommendations

Based on the findings, this study provides the following policy recommendations.

#### Prioritize socially supportive spatial design

Prioritise designing spaces that encourage meaningful social interaction, like multi-purpose communal areas, semi-private lounges, and outdoor gathering spaces. This study found a direct link between social design and mental health. This suggests that the way spaces are arranged is very important for helping older people connect with others, feel less lonely, and be happier with their lives.

#### Integration of environmental quality standards into CCRC regulations

Regulatory frameworks for eldercare facilities should include minimum standards for environmental quality, such as how much natural light, sound comfort, air quality, and access to greenery there is. These features were found to greatly improve how comfortable people thought they were, which is a sign of a good environmental appraisal and emotional stability.

#### Integrating evidence-based design into policy and practice

National and local plans for elder care should encourage the use of evidence-based design (EBD) frameworks when planning and evaluating CCRCs. Design evaluation criteria should go beyond just looking at how well a design works in terms of clinical safety and efficiency. They should also look at how it affects mental health, emotional satisfaction, and social connectedness.

### Limitations and future research directions

This study has several limitations. First, the study uses self-reported data, which may be biased, especially in older people. Second, the cross-sectional design makes it hard to figure out what caused what; longitudinal or experimental designs would be needed to prove that mediation effects last over time. Third, even though the sample comes from many CCRCs, it is mostly from eastern China, which may make it less applicable to other areas or cultures. Fourth, the fact that perceived comfort didn't have a significant effect may be because there were other variables that weren't measured, like a sense of belonging or social identity, that weren't included in the current model. Longitudinal studies should be used in future research to investigate how environmental factors and comfort perceptions change over time and how they affect mental health over time. Adding factors like autonomy, social identity, or purpose in life to the model might help us understand well-being in eldercare settings better. It would also be helpful to do qualitative studies on how older people understand and experience comfort, privacy, and social design. These studies would help us understand how these effects happen. Comparative studies of different types of cultures or facilities (like public vs. private CCRCs) may also show how cultural values and institutional structure affect how people see the built environment.

## Supplementary Information

Supplementary Material 1.

## Data Availability

The necessary data for the research findings may be obtained by submitting a written request to the grant funder.
