# Supplementary material for: Environmental quality, functional control, and social design as determinants of perceived comfort and psychological well-being among older residents in Chinese continuing care retirement communities
Source: BMC Geriatr. 2026 Apr 21;26:783. doi: 10.1186/s12877-026-07473-z (PMC13227821; doi:10.1186/s12877-026-07473-z)
Supplement: Supplementary file 1 — Supplementary Material 1. [file 12877_2026_7473_MOESM1_ESM.docx]

| Characteristics |
| --- |
| **Age (years)** |
| Below 60 |
| 60-69 |
| 70-79 |
| 80-89 |
| 90 and above |
| **Gender** |
| Male |
| Female |
| **Marital status** |
| Single |
| Married |
| Divorce |
| Widowed |
| **Level of education** |
| No education |
| Elementary school |
| Middle school |
| High school |
| University |
| Master's degree or higher |
| **Health status** |
| Excellent |
| Good |
| Fair |
| Poor |
| **Length of residence** |
| Less than half a year |
| More than half a year |
| **Living arrangement** |
| Alone |
| With spouse/partner |
| With other residents |

| **Construct/Associated Items** | **Factor loadings** | **CR** | **AVE** |
| --- | --- | --- | --- |
| **Functional Control** | | **0.96** | **0.877** |
| **Sense of control (SC)** |  |  |  |
| 1. Able to open and close doors | 0.892 |  |  |
| 2. Able to control the position and orientation of the bed | 0.875 |  |  |
| 3. Able to adjust the lighting intensity | 0.860 |  |  |
| 4. Able to control the volume of the TV and music | 0.894 |  |  |
| 5. Able to control the room temperature and humidity | 0.854 |  |  |
| 6. Able to open or close windows | 0.874 |  |  |
| 7. Able to control the call button | 0.871 |  |  |
| 8. Able to easily find your way or destination | 0.879 |  |  |
| 9. Able to personalize your room (e.g. placing family photos) | 0.834 |  |  |
| **Safety (SA)** | |  |  |
| 1. Having handrails (e.g. having bars in the bathroom/toilet of your room) | 0.853 |  |  |
| 2. Having proper non-slip flooring | 0.856 |  |  |
| 3. Having a safe storage area to stow personal belongings | 0.851 |  |  |
| 4. Having proper arrangement and location of furniture, clear and uncluttered pathways | 0.862 |  |  |
| 5. Having visual connection between rooms and nursing stations | 0.851 |  |  |
| **Social Design** | | **0.97** | **0.89** |
| **Social support (SS)** |  |  |  |
| 1. Having areas to facilitate communication and interaction (e.g. social activities and entertainment areas) | 0.785 |  |  |
| 2. Having comfortable and movable furniture (e.g. comfortable and movable chairs) | 0.786 |  |  |
| 3. Having areas for families and friends to stay overnight | 0.794 |  |  |
| **Privacy (PR)** | |  |  |
| 1. Having a single bedroom | 0.827 |  |  |
| 2. Having areas for private conversation | 0.820 |  |  |
| 3. Having visual privacy (e.g. having curtains and partitions) | 0.851 |  |  |
| **Environmental Quality** | | **0.96** | **0.88** |
| **Colour (CO)** |  |  |  |
| 1. Bright colours (e.g. light green, light yellow, white) | 0.885 |  |  |
| 2. Natural colours (e.g. colour of plants, sky, earth and sea) | 0.911 |  |  |
| 3. Warm colours (e.g. red, orange, yellow) | 0.869 |  |  |
| 4. Cool colours (e.g. green, blue, purple) | 0.909 |  |  |
| 5. Vibrant colours (e.g. vivid blue, purple) | 0.866 |  |  |
| 6. Harmonious colours (e.g. monochromatic, complementary, analogous, and split complementary colors) | 0.869 |  |  |
| 7. Chinese traditional colours (e.g. the following 12 Chinese traditional colours) | 0.902 |  |  |
| **Nature (NA)** |  |  |  |
| 1. Having natural materials (e.g. wooden flooring and furniture) | 0.749 |  |  |
| 2. Having natural sounds (e.g. sound of birds, water, breeze) | 0.819 |  |  |
| 3. Having water features (e.g., fountains, ponds, waterfalls) | 0.856 |  |  |
| 4. Having fresh air | 0.778 |  |  |
| 5. Having access to nature like flowers, plants, and trees | 0.783 |  |  |
| 6. Having window views of nature such as flowers, plants, trees, and the sky. | 0.734 |  |  |
| 7. Having a view of nature | 0.743 |  |  |
| **Art (AR)** |  |  |  |
| 1. Having nature-based artwork (e.g. paintings or photographs of natural scenery) | 0.839 |  |  |
| 2. Having areas for art viewing (e.g. areas for painting, calligraphy exhibitions) | 0.847 |  |  |
| 3. Having areas for artistic activities (e.g. areas for dancing, singing, painting) | 0.814 |  |  |
| 4. Having Chinese culture-relevant artworks (e.g. Chinese traditional painting, traditional patterned porcelain, and paper cutting) | 0.816 |  |  |
| **Lighting (LI)** |  |  |  |
| 1. Having adequate artificial light | 0.744 |  |  |
| 2. Having plenty of daylight | 0.782 |  |  |
| 3. Having low-positioned sensor night lights along the route used for getting up at night | 0.783 |  |  |
| **Noise control (NC)** |  |  |  |
| 1. Your room is removed from noise-producing areas | 0.794 |  |  |
| 2. Using silent machines in your room | 0.781 |  |  |
| 3. Your room is soundproofed | 0.838 |  |  |
| **Perceived Comfort** | | **0.98** | **0.89** |
| 1. My living space feels calm and peaceful. | 0.853 |  |  |
| 2. I can move around my private and communal spaces without difficulty. | 0.856 |  |  |
| 3. The temperature in my room is comfortable throughout the day. | 0.851 |  |  |
| 4. I am satisfied with the amount of natural light entering my room. | 0.862 |  |  |
| 5. The furniture provided is comfortable and meets my physical needs. | 0.851 |  |  |
| 6. The overall noise level allows me to rest and feel relaxed. | 0.881 |  |  |
| 7. I feel physically safe in both my room and shared areas. | 0.785 |  |  |
| 8. The colors and interior design help me feel emotionally at ease. | 0.786 |  |  |
| 9. I have adequate personal space to maintain privacy and comfort. | 0.794 |  |  |
| 10. The environment helps me feel emotionally stable and less anxious. | 0.853 |  |  |
| 11. Bathroom and hygiene facilities are clean and refreshing. | 0.862 |  |  |
| 12. I can control aspects of my room environment (e.g., lighting, ventilation). | 0.851 |  |  |
| 13. Communal areas (e.g., lounges, gardens) feel inviting and homely. | 0.881 |  |  |
| 14. I enjoy spending time in the physical surroundings of this community. | 0.785 |  |  |
| 15. The environment helps me feel connected to nature or the outdoors. | 0.786 |  |  |
| 16. Overall, this place feels like a comfortable home to me. | 0.889 |  |  |
| **Psychological Well‑Being Scale** | | **0.97** | **0.90** |
| 1. I am not afraid to voice my opinions, even when they are in opposition to the opinions of most people. | 0.862 |  |  |
| 2. My decisions are not usually influenced by what everyone else is doing. | 0.851 |  |  |
| 3. I tend to worry about what other people think of me. *(reverse)* | 0.881 |  |  |
| 4. In general, I feel I am in charge of the situation in which I live. | 0.785 |  |  |
| 5. The demands of everyday life often get me down. *(reverse)* | 0.786 |  |  |
| 6. I am quite good at managing the many responsibilities of my daily life. | 0.862 |  |  |
| 7. I think it is important to have new experiences that challenge how you think about yourself and the world. | 0.851 |  |  |
| 8. I have a sense that I have developed a lot as a person over time. | 0.881 |  |  |
| 9. I do not enjoy being in new situations that require me to change my old familiar ways of doing things. *(reverse)* | 0.785 |  |  |
| 10. People would describe me as a giving person, willing to share my time with others. | 0.786 |  |  |
| 11. I have not experienced many warm and trusting relationships with others. *(reverse)* | 0.862 |  |  |
| 12. Maintaining close relationships has been difficult and frustrating for me. *(reverse)* | 0.851 |  |  |
| 13. Some people wander aimlessly through life, but I am not one of them. | 0.862 |  |  |
| 14. I have a sense of direction and purpose in life. *(if original short form)* | 0.851 |  |  |
| 15. I sometimes feel as if I’ve done all there is to do in life. *(reverse)* | 0.881 |  |  |
| 16. I like most parts of my personality. | 0.785 |  |  |
| 17. When I look at the story of my life, I am pleased with how things have turned out so far. | 0.851 |  |  |
| 18. In many ways I feel disappointed about my achievements in life. *(reverse)* | 0.881 |  |  |
